# Childhood socio-economic disadvantages versus adverse care experiences: Mediation and moderation impacts on late-life depressive symptoms

**DOI:** 10.1192/j.eurpsy.2024.1760

**Published:** 2024-08-27

**Authors:** Ying Yue Huang, Wei Sen Zhang, Chao Qiang Jiang, Feng Zhu, Ya Li Jin, Shiu Lun Au Yeung, Jiao Wang, Kar Keung Cheng, Tai Hing Lam, Lin Xu

**Affiliations:** 1 School of Public Health, Sun Yat-sen University, Guangzhou, China; 2 Guangzhou Twelfth People’s Hospital, Guangzhou, China; 3 School of Public Health, The University of Hong Kong, Hong Kong, China; 4 Institute of Applied Health Research, University of Birmingham, Birmingham, UK; 5 Greater Bay Area Public Health Research Collaboration, China

**Keywords:** adverse care experiences, childhood socio-economic disadvantages, late-life depressive symptoms, mediation, moderation

## Abstract

**Background:**

Whether material deprivation-related childhood socio-economic disadvantages (CSD) and care-related adverse childhood experiences (ACE) have different impacts on depressive symptoms in middle-aged and older people is unclear.

**Methods:**

In the Guangzhou Biobank Cohort Study, CSD and ACE were assessed by 7 and 5 culturally sensitive questions, respectively, on 8,716 participants aged 50+. Depressive symptoms were measured by 15-item Geriatric Depression Scale (GDS). Multivariable linear regression, stratification analyses, and mediation analyses were done.

**Results:**

Higher CSD and ACE scores were associated with higher GDS score in dose-response manner (P for trend <0.001). Participants with one point increment in CSD and ACE had higher GDS score by 0.11 (95% confidence interval [CI], 0.09–0.14) and 0.41 (95% CI, 0.35–0.47), respectively. The association of CSD with GDS score was significant in women only (P for sex interaction <0.001; women: β (95% CI)=0.14 (0.11–0.17), men: 0.04 (−0.01 to 0.08)). The association between ACE and GDS score was stronger in participants with high social deprivation index (SDI) (P for interaction = 0.01; low SDI: β (95% CI)=0.36 (0.29–0.43), high SDI: 0.64 (0.48–0.80)). The proportion of association of CSD and ACE scores with GDS score mediated via education was 20.11% and 2.28%.

**Conclusions:**

CSD and ACE were associated with late-life depressive symptoms with dose-response patterns, especially in women and those with low adulthood socio-economic status. Education was a major mediator for CSD but not ACE. Eliminating ACE should be a top priority.

## 1. Introduction

Depression has become increasingly common in older people with heavy disease burden [1]. A 2021 meta-analysis by Tang et al. showed that the prevalence of depressive symptoms in adults aged ≥60 years in mainland China was 20%, and the prevalence increased with age [2]. A 2020 systematic review by Worrall et al. showed that health behaviours and socio-economic status (SES) were associated with depressive symptoms in older people, but the study did not consider childhood variables [3]. Similar to this systematic review, most reports were on later-life behavioural, social, and health status, while childhood variables had not been included. A 2017 meta-analysis by Nelson et al. showed that childhood maltreatment was a risk factor for depressive symptoms in older adults [4]. However, most of the studies in this meta-analysis were from high-income Western countries, and none from low-to-middle-income countries. Moreover, this meta-analysis examined direct childhood adversity and ignored indirect childhood adversity such as household difficulties [4]. A 2021 meta-analysis by Hughes et al. showed that childhood adversity, including both direct (e.g., maltreatment) and indirect (e.g., household difficulties) types, increased the risk of depressive symptoms in older adults [5]. However, this meta-analysis included only European countries and assumed that each type of childhood adversity had the same adverse effect on health. Note that the magnitude of the associations above may vary across socio-economic and political contexts [6, 7]. Whether these associations exist in other settings and ethnic groups and whether different types of childhood adversity have different impacts on depressive symptoms have not been reported.

Before and during the early years of the People’s Republic of China, most older Chinese people experienced harsh social and family environments during their childhood. During adulthood due to the open-door policy started about 40+ years ago, their livelihood and SES have continued to improve greatly. Such changes are quite different from people born in the same period in developed Western countries. Therefore, examining the associations between childhood adversity and late-life depressive symptoms in China may provide new insights for our understanding of depressive symptoms and early life risk factors.

Several conceptual models have been introduced to explain the associations of childhood adversity with late-life depressive symptoms, suggesting that adulthood socio-economic and health-related factors might be involved in the pathway. Nevertheless, the effect modifiers and underlying mechanisms remain unclear [8]. Moreover, most previous European studies used a cumulative measure of childhood adversities [5], which could not separate potentially differential effects of different types of childhood adversities on depressive symptoms [7, 9, 10]. Hence, in the present study, we used data from the Guangzhou Biobank Cohort Study (GBCS) to examine the associations of different types of childhood adversities separately, including material deprivation-related childhood socio-economic disadvantages (CSD) and care-related adverse childhood experiences (ACE), with depressive symptoms in middle-aged and older people, and potential moderation effect of sex, SES, and chronic diseases and mediations by socio-economic factors, health behaviours and stressful life events (SLE) in adulthood. We hypothesised that the number of CSD items and ACE items were positively associated with depressive symptoms in older people, and the associations, if any, might differ by sex and SES and involve different pathways.

## 2. Materials and methods

### 2.1. Study participants

The GBCS is a three-way collaboration among the Guangzhou Twelfth People’s Hospital and the Universities of Hong Kong, China, and Birmingham, UK. Details of the GBCS have been described previously [11]. Briefly, participants were recruited from the Guangzhou Health and Happiness Association for the Respective Elders, which is a community social and welfare organization with branches in all 10 districts of Guangzhou. Permanent residents in Guangzhou aged 50 years or above were eligible to participate. The baseline examination included a face-to-face interview by trained nurses using a computer-assisted standardized questionnaire. The study was approved by the Guangzhou Medical Ethics Committee of the Chinese Medical Association, and all participants provided written informed consent prior to participation. In phase 3 (2006–2008), the questionnaire included the validated Chinese version of the 15-item Geriatric Depression Scale (GDS) [12]; thus, in the present study, participants from phase 3 were included.

### 2.2. Exposures

CSD and ACE were exposure variables. Given the specific socio-historical context of China during the mid-20th century, standard tools for measuring CSD and ACE may not fully capture the range of experiences relevant to our study population. Therefore, we used measures developed from sociological accounts and prior research relevant to this context. Although these measures have been used in our previous studies, we acknowledge that they are not widely validated, which may limit the direct comparability of our findings.

We took into account parental possession and childhood material deprivation in CSD measurement. Parental possession included three simple and easily notable items, that is, a bicycle, a sewing machine, and a watch, based on sociological accounts of life in southern China in the mid-20th century and were used in our previous GBCS papers [13, 14]. Each item was coded as 0 for the present or 1 for the absent. Childhood material deprivation was assessed by four questions: “Did you usually have shoes when you were a child?,” “Did you usually get new clothes at Chinese New Year?,” “How often do you remember being hungry as a child?” and “How often did you eat meat as a child?” Each item was coded as zero when the answer was “Yes,” “Yes,” “Never,” and “Daily” for the four questions above, respectively, or as one otherwise. Then the cumulative CSD score was calculated. The CSD score ranged from 0 to 7, with higher CSD scores indicating greater CSDs. Participants were further classified into two categories as low CSD (CSD score < 4) and high CSD (CSD score ≥ 4) based on the median CSD score of 4.

ACE was assessed by the following five culturally sensitive questions before the age of 18 years as we reported previously [13, 15]: separation from mother for more than one year continuously, an experience so frightening as to be thought about years afterwards, being sent away from home because of wrongdoing, frequent quarrelling of parents, and early parental death. One point was assigned for a positive response of each question and zero point otherwise. The cumulative ACE score was calculated. The ACE score ranged from 0 to 5, with higher ACE score indicating more care-related ACEs. Participants were further classified into two categories as absence of ACE (ACE score = 0) and presence of ACE (ACE score ≥ 1) based on the median ACE score of 0.

### 2.3. Outcomes

The main outcome was the score of the 15-item GDS [12]. GDS was analysed as a continuous score, with higher scores indicating more negative symptoms. We also dichotomized the variable into the presence or absence of depressive symptoms as another outcome. The presence of depressive symptoms was defined by a GDS score of 8 or more, which is the standard cut-off score for the Chinese population [16] and has been widely used elsewhere, and reported in our previous papers [17–19].

### 2.4. Potential confounders, mediators, and effect modifiers

Sex and age (in years) were included as potential confounders in regression model 1 (main model). To further examine potential mediators of the associations of CSD and ACE with GDS score, we included socio-economic factors, health behaviours and SLE in adulthood in regression model 2. Socio-economic factors included education (primary or below, secondary, and college or above); occupation (manual, non-manual, and others); marital status (never married, married, separated, and widowed); and household income (<30 000 CNY/year, ≥30 000 CNY/year, and not known; US$1 = 7 CNY). Health behaviours included smoking status (never, former, and current smoker) and alcohol drinking status (never, former, and current user); physical activity (inactive, moderate, and active); and body mass index (BMI) (continuous variable). SLE in adulthood were defined as at least one of ten major life events in the last year, including separation or divorce, unemployment or retirement, business bankruptcy, physical assault, major conflict within family, major injury or traffic accident, death of spouse, major illness or death of a close family member, major natural disaster (such as flood or drought), and loss of all sources of income or living on debt, as reported in our previous papers [13, 20]. Moreover, CSD and ACE were also mutually adjusted in the regression model 3.

As women [2, 21], those with greater social deprivation [22, 23] and with chronic diseases [24] might be more vulnerable to depressive symptoms, sex, SES in adulthood, and history of chronic diseases were considered as potential effect modifiers. According to previous studies [25, 26], we derived a social deprivation index (SDI) as proxy for adult SES by summing the presence of the following four indicators, with one point assigned to each: never married status, primary school or below, unemployment, and household income < 30 000 CNY/year. The SDI score ranged from 0 to 4, with higher SDI scores indicating greater social deprivation and lower SES. Participants were further classified into low SDI (score 0–1) and high SDI (scores 2–4) based on half of the maximum SDI score. History of chronic diseases was defined by the presence of any of the following 20 diseases: hypertension, dyslipidaemia, type 2 diabetes mellitus, coronary heart disease, stroke, angina, rheumatic heart disease, arrhythmia, heart failure, cancer, liver disease, gastrointestinal disease, chest disease, genitourinary disease, neurological disease, eye disease, arthritis, thyroid disease, fracture history, and mental disease [27].

### 2.5. Statistical analysis

The chi-square test and analysis of variance were used, respectively, to compare the characteristics of categorical and continuous variables according to low/high CSD (<4 or ≥4) and ACE (0 or ≥1) score. Multivariable linear regression and logistic regression were used to analyse the associations of CSD and ACE with GDS score and the presence of depressive symptoms, respectively, giving adjusted regression coefficients (βs), odds ratios (ORs), and 95% confidence intervals (CIs). Multivariable linear regression was also used to analyse the associations of each CSD and ACE item with the GDS score. To analyse the potential moderation effect, interaction terms by multiplying CSD or ACE score and potential effect modifiers were generated, and the heterogeneity of models with and without interaction terms was compared. If a moderation effect exists, the interaction term would be statistically significant [28]. When a significant interaction was found, we conducted stratification analyses. To estimate the contribution of potential mediators to the association of CSD and ACE score with GDS score, we used causal mediation analysis under the counterfactual framework, which can decompose the averaged total effect into indirect effect (average causal mediation effect) and direct effect (average direct effect) [29]. For mediation analyses, potential mediators were dichotomized, that is, education (secondary or above vs. primary or below), occupation (unemployment vs. employment), marital status (never married vs. married), household income (≥30 000 CNY/year vs. <30 000 CNY/year), smoking status (ever vs. never), alcohol drinking status (ever vs. never), physical activity (moderate or above vs. inactive), and SLE in adulthood (yes vs. no). The “medeff” package in STATA was used to perform mediation analysis. All analyses were performed using STATA (Version 16.0; StataCorp LP, College Station, TX, USA). All tests were two-sided, and statistical significance was indicated by P < 0.05.

## 3. Results

### 3.1. Characteristics of participants

**Figure 1.**
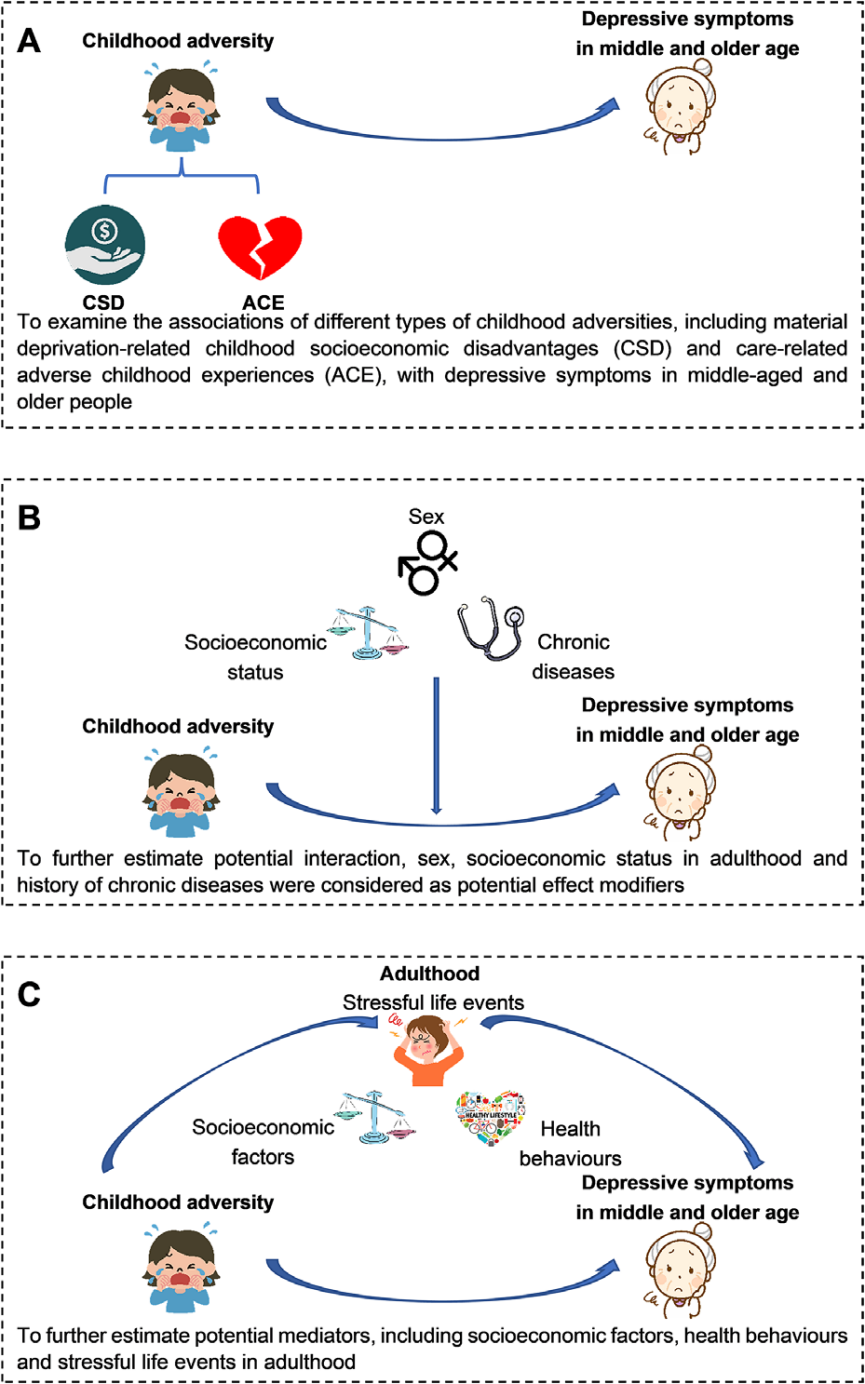
Overview of the present study models.

Of 10 088 participants recruited from 2006 to 2008, after excluding those with duplicate information (N=39), and missing information on CSD (N=353), ACE (N=687), GDS score (N=97), and potential mediators (N=429), 8 716 participants (86.4%) were included in the current study. Figure 1 shows an overview of the present study models.

Table 1 shows that participants with high CSD or ACE score had greater GDS score and higher prevalence of depressive symptoms (all P < 0.001). Participants with high CSD score were older, had higher proportions of men and current smokers, and had higher ACE score (all P < 0.001). They had lower proportions of married people, current alcohol users and those with SLEs in adulthood, and lower socio-economic position (lower education and household income and with manual occupation) (P from <0.001 to 0.003). Participants with high ACE score were also older, had higher proportions of men and current smokers, had higher CSD score but more with SLEs in adulthood (P from <0.001 to 0.01). They had lower proportions of married people and those with lower education and manual occupation, and lower household income (P from <0.001 to 0.04). No significant differences were found for physical activity and BMI (P from 0.07 to 0.70).

**Table 1.** Characteristics of the study sample by childhood socio-economic disadvantages or adverse childhood experiences

	Number of childhood socio-economic disadvantage (CSD) items (score)		*P*-value	Number of adverse childhood experience (ACE) items (score)		*P*-value
	<4	≥4		0	≥1	
Number of participants (row percentage)	3 331 (38.22%)	5 385 (61.78%)		4 822 (55.32%)	3 894 (44.68%)	
Sex, % men	20.41	28.38	<0.001	22.40	28.97	<0.001
Age, years, mean (SD)	57.33 (6.27)	62.21 (7.77)	<0.001	59.07 (7.02)	61.93 (8.01)	<0.001
Education (%)						
Primary or below	18.43	48.64	<0.001	34.22	40.65	<0.001
Secondary	69.35	45.24		57.55	50.62	
College or above	12.22	6.13		8.23	8.73	
Occupation (%)						
Manual	55.36	66.57	<0.001	63.40	60.91	0.04
Non–manual	26.27	17.44		19.97	21.85	
Others	18.37	15.99		16.63	17.23	
Marital status (%)						
Never married	0.93	0.54	<0.001	0.75	0.62	<0.001
Married	87.69	80.72		86.21	79.89	
Separated	1.98	1.26		1.31	1.82	
Widowed	9.40	17.47		11.74	17.67	
Household income, CNY/year (%)						
<30,000	29.48	39.35	<0.001	34.26	37.21	0.001
≥30,000	60.25	41.00		50.08	46.22	
Don’t know	10.27	19.65		15.66	16.56	
Smoking status (%)						
Never	86.52	79.78	<0.001	84.32	79.92	<0.001
Former	5.16	9.17		6.18	9.45	
Current	8.32	11.05		9.50	10.63	
Alcohol drinking (%)						
Never	51.91	55.06	<0.001	54.65	52.88	0.06
Former	4.77	5.81		4.96	5.98	
Current	43.32	39.13		40.40	41.14	
Physical activity (%)						
Inactive	7.87	7.86	0.62	7.55	8.24	0.07
Moderate	27.98	27.04		26.67	28.30	
Active	64.15	65.11		65.78	63.46	
Body mass index, kg/m^2^, mean (SD)	23.84 (3.29)	23.81 (3.36)	0.70	23.86 (3.40)	23.77 (3.26)	0.19
Stressful life events in adulthood (%)						
No	89.34	91.29	0.003	91.25	89.68	0.01
Yes	10.66	9.71		8.75	10.32	
ACE/CSD score, mean (SD)	0.50 (0.74)	0.71 (0.86)	<0.001	3.50 (2.02)	4.05 (1.94)	<0.001
GDS score, mean (SD)	2.19 (2.08)	2.57 (2.35)	<0.001	2.17 (2.09)	2.74 (2.42)	<0.001
Depressive symptoms (%)						
Absent (GDS <8)	97.42	95.06	<0.001	97.10	94.56	<0.001
Present (GDS ≥8)	2.58	4.94		2.90	5.44	

Abbreviations: GDS, Geriatric Depression Scale: higher scores indicating more negative symptoms; SD, standard deviation; US$1, 7 CNY.

### 3.2. Childhood adversities and GDS score in adulthood

Table 2 shows that higher CSD and ACE scores were associated with higher GDS score after adjusting for sex and age, with significant dose-response patterns (all P for trend <0.001). Participants with one point increment in CSD had GDS score increased by 0.11 (95% CI, 0.09–0.14) after adjusting for sex and age (Model 1). Moreover, GDS score increased by 0.41 (95% CI, 0.35 to 0.47) per ACE score (Model 1). After additionally adjusting for potential mediators and ACE or CSD, almost all the results remained significant with slightly attenuated associations (Models 2 and 3). Each item of CSD and ACE was associated with GDS score (Supplementary Tables S1 and S2). Of the CSD items, the associations of new clothes at Chinese New Year and hungry with GDS score appeared stronger than other items (adjusted mean differences, β (95% CI): 0.49 (0.39–0.59) and 0.43 (0.32–0.54), respectively) (Model 1). Of the ACE items, the associations of frightening experience thought about years afterwards, sent away from home because of wrongdoing and parents quarrelling frequently with GDS score were stronger than other items (β (95% CI): 0.97 (0.81–1.13), 0.88 (0.55–1.20), and 0.98 (0.80–1.16), respectively) (Model 1). The mean differences for these three ACE items were also greater than the seven CSD items. After additionally adjusting for potential mediators and ACE or CSD, the associations of CSD items with GDS score attenuated greatly, while the associations of ACE items with GDS score attenuated slightly (Models 2 and 3). Moreover, higher CSD and ACE scores were also associated with higher odds of depressive symptoms (all P for trend <0.001), and the ORs per ACE score were greater than those per CSD score (Models 1–3) (Supplementary Table S3).

**Table 2.** Associations of childhood socio-economic disadvantages and adverse childhood experiences with GDS score in adulthood

	*N*	Adjusted mean differences β (95% CI) in GDS score		
		Model 1	Model 2	Model 3
Number of childhood socio–economic disadvantage (CSD) items (score)				
0	517	Ref. (0)	Ref. (0)	Ref. (0)
1	1 215	0.17 (−0.06 to 0.40)	0.11 (−0.11 to 0.34)	0.11 (−0.11 to 0.34)
2	751	0.42 (0.17–0.67)^**^	0.33 (0.08–0.58)^**^	0.30 (0.05–0.54)*
3	848	0.37 (0.13–0.62)^**^	0.27 (0.02–0.51)*	0.24 (0.003–0.48)*
4	2 085	0.38 (0.17–0.60)^**^	0.23 (0.02–0.45)*	0.20 (−0.02 to 0.41)
5	1 469	0.75 (0.52–0.98)^***^	0.53 (0.30–0.76)^***^	0.45 (0.22–0.67)^***^
6	1 091	0.69 (0.45–0.93)^***^	0.45 (0.21–0.69)^***^	0.39 (0.16–0.63)^**^
7	740	0.84 (0.58–1.09)^***^	0.56 (0.30–0.82)^***^	0.45 (0.19–0.70)^**^
Per CSD score	8 716	0.11 (0.09–0.14)^***^	0.07 (0.05–0.10)^***^	0.06 (0.03–0.08)^***^
P for trend		<0.001	<0.001	<0.001
Number of adverse childhood experience (ACE) items (score)				
0	4 822	Ref. (0)	Ref. (0)	Ref. (0)
1	2 592	0.40 (0.29–0.51)^***^	0.40 (0.29 to 0.50)^***^	0.38 (0.28 to 0.49)^***^
2	1 040	0.70 (0.55–0.85)^***^	0.66 (0.51 to 0.81)^***^	0.64 (0.49 to 0.79)^***^
3	235	1.26 (0.97–1.56)^***^	1.15 (0.86 to 1.44)^***^	1.11 (0.82 to 1.40)^***^
4	26	2.74 (1.88–3.60)^***^	2.61 (1.76 to 3.45)^***^	2.56 (1.72 to 3.40)^***^
5	1	11.70 (7.33–16.08)^***^	11.62 (7.33 to 15.91)^***^	11.50 (7.22 to 15.78)^***^
Per ACE score	8 716	0.41 (0.35–0.47)^***^	0.38 (0.33–0.44)^***^	0.37 (0.31–0.43)^***^
P for trend		<0.001	<0.001	<0.001

Abbreviations: CI, confidence interval; GDS, Geriatric Depression Scale: higher scores indicating more negative symptoms; N, number; Ref, reference.

*Note:* Model 1: adjusting for sex, and age. Model 2: additionally adjusting for education, occupation, marital status, household income, smoking, alcohol drinking, physical activity, body mass index, and stressful life events in adulthood. Model 3: additionally adjusting for adverse childhood experiences (ACE score) or childhood socio-economic disadvantages (CSD score).

* *P* < 0.05,

** *P* < 0.01,

*** *P* < 0.001.

### 3.3. Childhood adversities and GDS score in adulthood by sex

Table 3 shows a significant moderation effect of sex on the association between CSD score and GDS score in Model 1 (P for interaction < 0.001). Subgroup analyses by sex showed that the associations of CSD with GDS score became stronger with a significant trend (P < 0.001) in women. However, men showed no significant associations (except for those with two and seven items) and trend (P = 0.14). The GDS score increased by 0.14 (95% CI, 0.11–0.17) in women per CSD score, but the small increase in men was not significant. After additionally adjusting for potential mediators and ACE, the associations for CSD in men and women much attenuated (Models 2 and 3). Although no significant moderation effect of sex was observed for the association between ACE score and GDS score (P for interaction = 0.22 in Model 1), the associations of ACE score with GDS score also appeared to be stronger in women. After additionally adjusting for potential mediators and CSD, the results for ACE in men and women were similar (Models 2 and 3).

**Table 3.** Associations of childhood socio-economic disadvantages and adverse childhood experiences with GDS score in adulthood by sex

	*N*	Adjusted mean differences β (95% CI) in GDS score		
		Model 1	Model 2	Model 3
	Men			
Number of childhood socio–economic disadvantage (CSD) items (score)				
0	97	Ref. (0)	Ref. (0)	Ref. (0)
1	220	0.23 (−0.27 to 0.74)	0.11 (−0.38 to 0.61)	0.14 (−0.35 to 0.63)
2	167	0.73 (0.20–1.26)^**^	0.65 (0.14 to 1.17)*	0.67 (0.15 to 1.18)*
3	196	0.14 (−0.38 to 0.65)	−0.02 (−0.53 to 0.48)	0.01 (−0.49 to 0.51)
4	513	0.29 (−0.17 to 0.75)	0.16 (−0.29 to 0.62)	0.15 (−0.30 to 0.60)
5	440	0.44 (−0.03 to 0.91)	0.24 (−0.23 to 0.70)	0.20 (−0.26 to 0.66)
6	339	0.30 (−0.18 to 0.78)	0.13 (−0.35 to 0.61)	0.11 (−0.37 to 0.58)
7	236	0.56 (0.06–1.07)*	0.35 (−0.15 to 0.85)	0.24 (−0.25 to 0.74)
Per CSD score	2 208	0.04 (−0.01 to 0.08)	0.01 (−0.03 to 0.06)	−0.003 (−0.05 to 0.04)
P for trend		0.14	0.58	0.90
	Women			
Number of childhood socio–economic disadvantage (CSD) items (score)				
0	420	Ref. (0)	Ref. (0)	Ref. (0)
1	995	0.15 (−0.11 to 0.42)	0.11 (−0.15 to 0.37)	0.10 (−0.15 to 0.36)
2	584	0.32 (0.03–0.61)*	0.23 (−0.05 to 0.51)	0.18 (−0.10 to 0.46)
3	652	0.43 (0.15–0.71)^**^	0.33 (0.05–0.61)*	0.29 (0.01–0.56)*
4	1 572	0.40 (0.15–0.65)^**^	0.24 (−0.003 to 0.49)	0.20 (−0.05 to 0.44)
5	1 029	0.85 (0.59–1.11)^***^	0.63 (0.36–0.89)^***^	0.52 (0.26–0.79)^***^
6	753	0.82 (0.54–1.10)^***^	0.55 (0.27–0.83)^***^	0.49 (0.21–0.77)^**^
7	504	0.92 (0.61–1.22)^***^	0.61 (0.31–0.92)^***^	0.51 (0.20–0.81)^**^
Per CSD score	6 508	0.14 (0.11–0.17)^***^	0.09 (0.06–0.12)^***^	0.08 (0.05–0.11)^***^
P for trend		<0.001	<0.001	<0.001
P for sex interaction		<0.001	0.004	0.002
	Men			
Number of adverse childhood experience (ACE) items (score)				
0	1 080	Ref. (0)	Ref. (0)	Ref. (0)
1	710	0.24 (0.04–0.44)*	0.24 (0.04–0.43)*	0.22 (0.03–0.42)*
2	334	0.60 (0.34–0.86)^***^	0.60 (0.34–0.86)^***^	0.59 (0.33–0.84)^***^
3	77	1.26 (0.77–1.75)^***^	1.14 (0.66–1.62)^***^	1.15 (0.67–1.63)^***^
4	7	2.21 (0.65–3.77)^**^	2.18 (0.66–3.71)^**^	2.17 (0.64–3.69)^**^
5	0	–	–	–
Per ACE score	2 208	0.35 (0.24 to 0.45)^***^	0.33 (0.23 to 0.43)^***^	0.33 (0.22–0.43)^***^
P for trend		<0.001	<0.001	<0.001
	Women			
Number of adverse childhood experience (ACE) items (score)				
0	3 742	Ref. (0)	Ref. (0)	Ref. (0)
1	1 882	0.46 (0.33–0.58)^***^	0.46 (0.34–0.59)^***^	0.44 (0.31–0.56)^***^
2	706	0.74 (0.56–0.93)^***^	0.70 (0.51–0.88)^***^	0.66 (0.48–0.84)^***^
3	158	1.27 (0.91–1.63)^***^	1.15 (0.79–1.51)^***^	1.10 (0.74–1.45)^***^
4	19	2.93 (1.90–3.95)^***^	2.77 (1.77–3.78)^***^	2.73 (1.73–3.74)^***^
5	1	11.69 (7.23–16.14)^***^	11.61 (7.24–15.98)^***^	11.45 (7.09–15.81)^***^
Per ACE score	6 508	0.43 (0.36–0.50)^***^	0.41 (0.34–0.48)^***^	0.39 (0.32–0.46)^***^
P for trend		<0.001	<0.001	<0.001
P for sex interaction		0.22	0.34	0.33

Abbreviations: CI, confidence interval; GDS, Geriatric Depression Scale: higher scores indicating more negative symptoms; N, number; Ref, reference.

*Note:* Model 1: adjusting for age. Model 2: additionally adjusting for education, occupation, marital status, household income, smoking, alcohol drinking, physical activity, body mass index, and stressful life events in adulthood. Model 3: additionally adjusting for adverse childhood experiences (ACE score) or childhood socio-economic disadvantages (CSD score).

* *P* < 0.05.

** *P* < 0.01.

*** *P* < 0.001.

### 3.4. Childhood adversities and GDS score in adulthood by SDI and chronic diseases

Table 4 shows no significant moderation effect of SDI on the association between CSD score and GDS score in Model 1 (P for interaction = 0.30), but when CSD score was dichotomized into good (CSD scores 0–3) and poor (CSD scores 4–7) childhood socio-economic conditions (Supplementary Table S4 Model 1), a significant moderation effect was found (P for interaction = 0.01). Those with poor childhood socio-economic conditions and high SDI in adulthood had the highest GDS score. A significant moderation effect of adulthood SDI on the ACE score and GDS score association was found (P for interaction = 0.01). Compared with participants with low SDI, the association between ACE score and GDS score was stronger in those with high SDI. The GDS score increased by 0.36 (95% CI, 0.29–0.43) per ACE score for low SDI, but by 0.64 (95% CI, 0.48–0.80) for high SDI. After additionally adjusting for potential mediators and ACE or CSD, the results were similar for per ACE score but much attenuated for per CSD score (Models 2 and 3). Chronic diseases did not significantly moderate the association of CSD/ACE with GDS score (P for interaction = 0.62 and 0.96, respectively, in Model 1) (table not shown).

**Table 4.** Associations of childhood socio-economic disadvantages and adverse childhood experiences with GDS score in adulthood by social deprivation index

	*N*	Adjusted mean differences β (95% CI) in GDS score		
		Model 1	Model 2	Model 3
	Low social deprivation index (SDI) in adulthood (0–1)			
Number of childhood socio–economic disadvantage (CSD) items (score)				
0	437	Ref. (0)	Ref. (0)	Ref. (0)
1	1 044	0.24 (0.009–0.48)*	0.20 (−0.03 to 0.43)	0.19 (−0.04 to 0.42)
2	587	0.48 (0.23–0.74)^***^	0.44 (0.18 to 0.69)^**^	0.40 (0.14 to 0.65)^**^
3	654	0.40 (0.14–0.65)^**^	0.32 (0.07–0.57)*	0.29 (0.04–0.54)*
4	1 451	0.33 (0.10–0.55)^**^	0.24 (0.01–0.46)*	0.19 (−0.04 to 0.41)
5	889	0.61 (0.37–0.85)^***^	0.49 (0.25–0.73)^***^	0.42 (0.17–0.66)^**^
6	587	0.51 (0.25–0.77)^***^	0.39 (0.13–0.66)^**^	0.34 (0.08–0.60)*
7	342	0.57 (0.27–0.87)^***^	0.45 (0.15–0.75)^**^	0.32 (0.01–0.62)*
Per CSD score	5 991	0.07 (0.04–0.09)^***^	0.05 (0.02–0.08)^**^	0.03 (0.01–0.06)*
P for trend		<0.001	0.001	0.02
	High social deprivation index (SDI) in adulthood (2–4)			
Number of childhood socio–economic disadvantage (CSD) items (score)				
0	29	Ref. (0)	Ref. (0)	Ref. (0)
1	68	−0.09 (−1.23 to 1.06)	−0.40 (−1.54 to 0.73)	−0.25 (−1.36 to 0.86)
2	73	0.07 (−1.06 to 1.20)	−0.19 (−1.31 to 0.93)	−0.11 (−1.20 to 0.98)
3	97	0.21 (−0.88 to 1.30)	0.16 (−0.92 to 1.25)	0.19 (−0.87 to 1.25)
4	317	0.41 (−0.59 to 1.41)	0.26 (−0.74 to 1.26)	0.32 (−0.65 to 1.30)
5	303	0.91 (−0.10 to 1.91)	0.68 (−0.32 to 1.69)	0.58 (−0.40 to 1.57)
6	243	0.65 (−0.36 to 1.66)	0.50 (−0.52 to 1.52)	0.47 (−0.53 to 1.46)
7	195	0.84 (−0.19 to 1.87)	0.70 (−0.34 to 1.73)	0.65 (−0.36 to 1.65)
Per CSD score	1 325	0.15 (0.07–0.24)^***^	0.16 (0.07–0.24)^***^	0.13 (0.04–0.21)^**^
P for trend		<0.001	<0.001	0.003
P for SDI interaction		0.30	0.12	0.16
	Low social deprivation index (SDI) in adulthood (0–1)			
Number of adverse childhood experience (ACE) items (score)				
0	3 428	Ref. (0)	Ref. (0)	Ref. (0)
1	1 749	0.39 (0.27–0.51)^***^	0.39 (0.27–0.51)^***^	0.37 (0.26–0.49)^***^
2	656	0.66 (0.49–0.84)^***^	0.64 (0.47–0.82)^***^	0.63 (0.45–0.80)^***^
3	141	1.11 (0.76–1.46)^***^	1.02 (0.67–1.37)^***^	0.99 (0.65–1.34)^***^
4	17	1.89 (0.90–2.88)^***^	1.70 (0.72–2.68)^**^	1.69 (0.71–2.67)^**^
5	0	–	–	–
Per ACE score	5 991	0.36 (0.29–0.43)^***^	0.35 (0.28–0.41)^***^	0.34 (0.27–0.40)^***^
P for trend		<0.001	<0.001	<0.001
Number of adverse childhood experience (ACE) items (score)				
0	639	Ref. (0)	Ref. (0)	Ref. (0)
1	415	0.47 (0.15–0.80)^**^	0.50 (0.19–0.82)^**^	0.47 (0.15–0.79)^**^
2	216	1.04 (0.64–1.44)^***^	1.00 (0.60–1.40)^***^	0.95 (0.55–1.35)^***^
3	49	2.08 (1.34–2.83)^***^	1.97 (1.24–2.71)^***^	1.88 (1.14–2.61)^***^
4	5	4.20 (1.93–6.46)^***^	4.23 (2.01–6.46)^***^	4.13 (1.91–6.35)^***^
5	1	11.38 (6.34–16.41)^***^	11.89 (6.94–16.84)^***^	11.69 (6.74–16.64)^***^
Per ACE score	1 325	0.64 (0.48–0.80)^***^	0.63 (0.47–0.79)^***^	0.60 (0.44–0.76)^***^
P for trend		<0.001	<0.001	<0.001
P for SDI interaction		0.01	0.008	0.008

Abbreviations: CI, confidence interval; GDS, Geriatric Depression Scale: higher scores indicating more negative symptoms; N, number; Ref, reference.

*Note:* Model 1: adjusting for sex, and age. Model 2: additionally adjusting for education, occupation, marital status, household income, smoking, alcohol drinking, physical activity, body mass index, and stressful life events in adulthood. Model 3: additionally adjusting for adverse childhood experiences (ACE score) or childhood socio-economic disadvantages (CSD score).

* *P* < 0.05.

** *P* < 0.01.

*** *P* < 0.001.

### 3.5. Mediation analyses

Table 5 shows that the association of CSD score with GDS score was partly mediated by education, household income and smoking status after adjusting for sex and age, and the proportion of mediation was 20.11% (95% CI, 15.88%–25.93%), 12.19% (95% CI, 9.32%–16.32%), and 2.17% (95% CI, 1.75%–2.72%), respectively (all P < 0.05). However, occupation, marital status, alcohol drinking status, physical activity, BMI, and SLE showed no mediation. For ACE, the proportions via mediation to GDS by education, physical activity and SLE in adulthood were significant but small, being 2.28% (95% CI, 1.98%–2.63%), 1.29% (95% CI, 1.12%–1.49%), and 1.72% (95% CI, 1.48%–1.97%), respectively. Alcohol drinking status (ever vs. never) showed a suppressive effect on the association of ACE score with adulthood GDS score (−1.14%, 95% CI, −1.31% to −0.99%). Occupation, marital status, household income, smoking status, and BMI showed no significant mediation.

**Table 5.** Associations of childhood socio-economic disadvantages and adverse childhood experiences with GDS score in adulthood with mediation by potential mediators

Mediators	Indirect effect (ACME) Estimate (95% CI)^a^	Direct effect (ADE) Estimate (95% CI)^a^	Total effect Estimate (95% CI)^a^	Proportion via mediation % (95% CI)^a^
Childhood socio–economic disadvantages				
Education (secondary or above vs. primary or below)	0.0208 (0.0157–0.0264)*	0.0832 (0.0592–0.1110)*	0.1040 (0.0802–0.1311)*	20.11 (15.88–25.93)*
Occupation (unemployment vs. employment)	−0.0001 (−0.0008 to 0.0005)	0.1123 (0.0891–0.1391)*	0.1122 (0.0890–0.1389)*	−0.08 (−0.09 to −0.06)
Marital status^b^ (never married vs. married)	−0.0011 (−0.0035 to 0.0002)	0.1129 (0.0884–0.1412)*	0.1118 (0.0878–0.1403)*	−0.99 (−1.25 to −0.79)
Household income^c^ (≥30,000 CNY/year vs. <30,000 CNY/year)	0.0116 (0.0084–0.0146)*	0.0843 (0.0593–0.1131)*	0.0959 (0.0712–0.1248)*	12.19 (9.32–16.32)*
Smoking (ever vs. never)	0.0025 (0.0004–0.0049)*	0.1111 (0.0879–0.1379)*	0.1136 (0.0904–0.1403)*	2.17 (1.75–2.72)*
Alcohol drinking (ever vs. never)	0.0007 (−0.0013 to 0.0029)	0.1119 (0.8873–0.1386)*	0.1125 (0.0891–0.1389)*	0.58 (0.47–0.74)
Physical activity (moderate or above vs. inactive)	0.0016 (−0.0014 to 0.0045)	0.1128 (0.0897–0.1395)*	0.1144 (0.0908–0.1408)*	1.42 (1.15–1.79)
Body mass index	−0.00004 (−0.0013–0.0012)	0.1124 (0.0892–0.1391)*	0.1123 (0.0890–0.1392)*	−0.04 (−0.05 to −0.03)
SLE in adulthood (yes vs. no)	−0.0014 (−0.0033 to 0.0001)	0.1131 (0.0900–0.1398)*	0.1117 (0.0887–0.1387)*	−1.29 (−1.62 to −1.04)
Adverse childhood experiences				
Education (secondary or above vs. primary or below)	0.0093 (0.0034–0.0157)*	0.3981 (0.3444–0.4603)*	0.4074 (0.3522–0.4689)*	2.28 (1.98 to 2.63)*
Occupation (unemployment vs. employment)	−0.0005 (−0.0026 to 0.0012)	0.4076 (0.3535–0.4700)*	0.4071 (0.3531–0.4691)*	−0.12 (−0.14 to −0.011)
Marital status^b^ (never married vs. married)	0.00004 (−0.0017 to 0.0019)	0.3838 (0.3254–0.4512)*	0.3838 (0.3261–0.4506)*	0.01 (0.01–0.01)
Household income^c^ (≥30,000 CNY/year vs. <30,000 CNY/year)	0.0051 (−0.0032 to 0.0131)	0.4249 (0.3674–0.4913)*	0.4300 (0.3715–0.4984)*	1.18 (1.02–1.37)
Smoking (ever vs. never)	0.0005 (−0.0019 to 0.0030)	0.4067 (0.3527–0.4690)*	0.4072 (0.3540–0.4695)*	0.12 (0.10–0.13)
Alcohol drinking (ever vs. never)	−0.0046 (−0.0097 to −0.0003)*	0.4120 (0.3581–0.4743)*	0.4074 (0.3526–0.4692)*	−1.14 (−1.31 to −0.99)*
Physical activity (moderate or above vs. inactive)	0.0053 (0.0010–0.0101)*	0.4039 (0.3500–0.4661)*	0.4092 (0.3544–0.4721)*	1.29 (1.12–1.49)*
Body mass index	0.0023 (−0.0003 to 0.0056)	0.4049 (0.3509–0.4672)*	0.4072 (0.3526–0.4701)*	0.57 (0.50–0.67)
SLE in adulthood (yes vs. no)	0.0070 (0.0030–0.0115)*	0.3990 (0.3450–0.4613)*	0.4059 (0.3525–0.4686)*	1.72 (1.48–1.97)*

Abbreviations: ACME, average causal mediated effect; ADE, average direct effect; CI, confidence interval; GDS, Geriatric Depression Scale; SLE, stressful life events.

a Adjusting for sex and age.

b Sample size = 7,328.

c Sample size = 7,316.

* *P* < 0.05.

## 4. Discussion

We have first reported that both material deprivation-related CSD and care-related ACE showed dose-response associations with depressive symptoms in middle to older age. The associations were stronger for ACE than CSD, in women and those with low adulthood SES, and education was the main and important mediator of the associations of CSD with GDS score (20% mediation) but was the main but small mediator of the associations of ACE with GDS score (2% mediation).

Our findings in a setting with social development patterning very different from Western populations are consistent with previous studies mostly from Western countries showing that childhood adversities were associated with late-life depressive symptoms with dose-response patterns [5, 30–32]. The associations of childhood adversities, mainly maltreatment or care-related ACE, with depressive symptoms assessed by the Short Form of the Center for Epidemiologic Studies Depression Scale were also reported by two recent Chinese studies [33, 34]. Note that consistent findings across different settings with different socio-economic and political contexts will provide more robust evidence to support causation. However, no previous studies distinguished and compared different types of childhood adversities in China and other countries. Previous Chinese studies just reported the associations of famine or deprivation [35, 36] or maltreatment or care-related ACE [33, 37, 38], or integrated childhood starvation or food deprivation and ACE into one variable [34, 39] with late-life depressive symptoms. Our study has shown new results that both material deprivation-related CSD and care-related ACE were associated with late-life depressive symptoms even after mutual adjustment. However, the associations for ACE were stronger than CSD, indicating that the deleterious effect of psychological adverse events might be greater than childhood poverty. Moreover, we have shown that the dose-response relationships persisted after adjusting for potential mediators, indicating that childhood adversities may have direct long-lasting effects on late-life depression. Hence, our results could help identifying children or adults at risk of depression at older age.

We have also first reported on the different results of individual CSD and ACE items. Among the CSD items, not receiving new clothes at Chinese New Year and experiencing hunger were particularly strong predictors of GDS scores. This might be attributed to the deep cultural importance of New Year traditions in Chinese society, where new clothes symbolize renewal and familial care, making the absence of such a tradition especially memorable. Additionally, hunger, being a direct threat to physical well-being, likely has a profound and lasting psychological impact, distinguishing these experiences from other forms of material deprivation such as the lack of shoes or less frequent meat consumption. Under ACE, the associations of frightening experience thought about years afterwards, having been sent away from home because of wrongdoing and parents quarrelling frequently with GDS score were the strongest, and stronger than the two CSD items above. However, while some studies, including a systematic review and meta-analysis by Simbi et al. [40], have highlighted early parental death as a risk factor for later-life depression, our study did not find a significant association between early parental death and GDS scores in our older adult population. As shown in Supplementary Table S1, the GDS score for middle-aged and older individuals with early parental death increased by 0.14 (95% CI, 0.02–0.26) after adjusting for sex and age in Model 1. However, this association was attenuated to null (β (95% CI): 0.12 (−0.0004 to 0.24)) after further adjustments for socio-economic factors, health behaviours, and SLEs in adulthood (Model 2). This non-significant result could be attributed to several factors, including the unique sociocultural and historical context of our study population, where the traditional structure of Chinese families might have provided additional support and resilience. Moreover, the meta-analysis by Simbi et al. [40] primarily focused on individuals aged 18–65, which might not fully represent the middle-aged and older populations in our study. The potential for the deleterious effects of early parental death on mental health to weaken over time due to the quality of other relationships and socio-economic positions in adulthood further complicates the direct comparison. This highlights the necessity of a nuanced approach in understanding the potential impact of early life adversities, taking into account the specific characteristics of the population under study and the multifaceted nature of depression.

Our findings could be explained by human brain development. Childhood is a key period when there are major advances in the brain to develop skills in learning, reasoning, and understanding, which are essential in subsequent social success [10]. Childhood adversity may lead to structural variation in brain grey and white matter, functional variation in brain activity and functional connectivity, and altered neurotransmitter metabolism or production, which could subsequently increase vulnerability to depression in adulthood [41]. Moreover, as young children have little awareness of social structures, psychosocial stress such as the feelings of inferiority, subordination, or lack of control might emerge mainly due to the adverse feelings and behaviours of their caregivers, which in turn could influence mental health via neuroendocrine pathways [42]. And needs theory suggests that once basic needs that can be bought with money are met, increasing levels of wealth do not add any more to the overall levels of happiness [43]. Thus, care-related ACE might be more harmful for mental health than material deprivation-related CSD.

Depression is more common among women than men [21], but whether the associations between childhood adversities and late-life depressive symptoms vary by sex has been inconclusive. Previous studies showed mixed results, with some reporting stronger associations in women [44–46], some reporting similar associations in men and women [33, 47, 48], and some reporting stronger associations in men [49, 50]. We have reported the first result that although men might have more CSD and ACE, the associations of childhood adversities, especially material deprivation-related CSD, with late-life depressive symptoms were much stronger in women. Deeply ingrained patriarchal traditions in China might explain the sex differences. Historically, daughters were treated as “lost investment” in China [51], which might result in unequal treatment and opportunities for women compared to men, leading to women with childhood poverty more vulnerable to depression due to the cumulative effects of societal discrimination, limited opportunities, and unequal access to resources. However, although we observed significant sex interactions in the association between CSD and GDS scores, such interactions were not evident between ACE and GDS scores. This finding suggests that the impact of ACE on depressive symptoms in later life may not differ markedly between men and women. We hypothesize that this could be due to the pervasive nature of ACEs, which often involve emotional and interpersonal dynamics that might equally affect individuals regardless of sex. Additionally, cultural and social norms surrounding gender roles and emotional expression could influence the reporting and processing of ACEs, potentially contributing to the observed results. Further studies exploring these cultural and social dimensions could provide deeper insights into the mechanisms underlying these associations.

Moreover, our results that the associations of childhood adversities, mainly care-related ACE, with late-life depressive symptoms also varied by adulthood SES, corroborate results of previous studies from Japan [52] and China Health and Retirement Longitudinal Study [38]. The Japanese study defined adult SES based on educational attainment and annual household income [52], and the Chinese study used annual per capita household consumption expenditure to indicate participants’ current economic status [38]. Both studies found that achieving high adulthood SES could ameliorate the adverse effects of childhood adversities, mainly psychological adverse events, on late-life depression. This might be explained by the social mobility model, suggesting that the adverse effects of childhood adversity may be mitigated or reversed by upward mobility, that is, improved SES in adulthood [53], due to less economic pressure and more access to health resources related to individuals with high adulthood SES [54]. Our study used a composite indicator, that is, SDI, to assess SES in adulthood, and suggested that high adult SES might also potentially mitigate the adverse effects of childhood poverty on late-life depression. However, the absence of a significant interaction between CSD and SDI warrants a cautious interpretation of the potential buffering effect of high adult SES. It is possible that factors not captured by our SDI, such as psychological resilience, social support, or access to mental health resources, may play critical roles in mitigating the impact of childhood poverty. Additionally, the uniform measure of SES represented by SDI may not fully capture the diverse aspects of SES and their nuanced effects on mental health outcomes.

Consistent with previous studies [34, 45, 55–57], our study found that education might act as a mediator against the adverse effects of childhood adversities on late-life depressive symptoms, indicating that expanding coverage of universal secondary education, including females equally, might be the most important intervention to reduce socio-economic disparities and late-life depression symptoms in people with childhood adversities. However, previous studies did not compare the mediating effects of education on different types of childhood adversities. We first found that the mediating effect of education was much greater for CSD (20%) than ACE (2%), and we also found that the association of CSD with depressive symptoms could be partly mediated by higher household income (10%). Because ACE showed strong and almost 100% direct effect with a very small proportion of effect via mediators, if such associations are causal, eliminating or reducing ACE and related psychological traumas in childhood should be a top priority to promote childhood mental health and prevent mental ill health in adult life.

Our study had some limitations. First, as information of CSD and ACE was collected by self-report like a case-cohort study, recall errors might have led to random and systemic errors. Random errors would result in underestimation of the strength of associations. Participants with depressive symptoms might have reported more childhood adversities than those without depressive symptoms. However, we used relatively objective and specific indicators to assess childhood adversities, such as parental possession and parental death, which might be hard to forget and have been supported in our previous papers [13–15]. Second, as our study used specific measures for CSD and ACE rather than a widely used and validated standard tool, direct comparability of our results with those of previous studies may be limited. However, it is worth noting that the items of CSD were tailored to mid-20th century China, based on sociological accounts of life in southern China during that era [14]. Similarly, the ACE items have been considered in other studies, including the National Population Health Survey of the Canadian population and the China Health and Retirement Longitudinal Study [58–60]. Third, we were unable to ascertain the timing of onset for depressive symptoms and other health conditions. But the timing of the data on childhood adversities should most likely precede depressive symptoms. Fourth, our CSD and ACE scores were self-reported subjective measures of cumulative childhood adversities [61]. Future studies using objective and documented childhood exposure data are warranted. Finally, underlying mechanisms through genetics, pathology, or biomarker-related factors were not examined, but such factors are unclear or unknown.

In conclusion, both material deprivation-related CSD and care-related ACE were associated with late-life depressive symptoms with dose-response patterns. The associations were stronger in women and those with low adulthood SES. Education was a major mediator for CSD but not ACE, highlighting the role of improving and equitable access to education in mitigating the adverse effects of CSDs. With rapid development in economy and popularization of basic compulsory education in China and many low- and middle-income countries, some CSD items could have been reduced but ACE might not. But eliminating care-related ACEs should be a top priority to prevent mental ill health in adulthood. Further studies are needed to clarify the mechanisms and examine the consequences of current CSD and ACE on future depression.

## Supporting information

Huang et al. supplementary material Huang et al. supplementary material

## Data Availability

Data that support findings are restricted to researchers who have permission from the Guangzhou Biobank Cohort Study, and so are not publicly available.

## Supplementary material

The supplementary material for this article can be found at http://doi.org/10.1192/j.eurpsy.2024.1760.
