# Supplementary material for: Childhood socio-economic disadvantages versus adverse care experiences: Mediation and moderation impacts on late-life depressive symptoms
Source: Eur Psychiatry. 2024 Aug 27;67(1):e47. doi: 10.1192/j.eurpsy.2024.1760 (PMC11441342; doi:10.1192/j.eurpsy.2024.1760)
Supplement: Huang et al. supplementary material [file S0924933824017607sup001.pdf]

Table S1. Associations of each childhood socioeconomic disadvantage item with GDS score in adulthood.

| | *N* | Adjusted mean differences β (95% CI) in GDS score | | |
|---|---|---|---|---|
| | | Model 1 | Model 2 | Model 3 |
| **Parental possession** | | | | |
| **Bicycle** | | | | |
| Yes | 2 617 | Ref. (0) | Ref. (0) | Ref. (0) |
| No | 6 099 | 0.24 (0.13 to 0.35)*** | 0.12 (0.01 to 0.23)* | 0.08 (-0.03 to 0.18) |
| **Sewing machine** | | | | |
| Yes | 2 818 | Ref. (0) | Ref. (0) | Ref. (0) |
| No | 5 898 | 0.32 (0.21 to 0.42)*** | 0.17 (0.07 to 0.28)** | 0.14 (0.03 to 0.24)* |
| **Watch** | | | | |
| Yes | 3 004 | Ref. (0) | Ref. (0) | Ref. (0) |
| No | 5 712 | 0.25 (0.14 to 0.35)*** | 0.10 (-0.002 to 0.21) | 0.07 (-0.04 to 0.17) |
| **Childhood material deprivation** | | | | |
| **Shoes** | | | | |
| Yes | 6 475 | Ref. (0) | Ref. (0) | Ref. (0) |
| No | 2 241 | 0.13 (0.02 to 0.24)* | 0.01 (-0.10 to 0.12) | -0.02 (-0.13 to 0.09) |
| **New clothes at Chinese New Year** | | | | |
| Yes | 5 776 | Ref. (0) | Ref. (0) | Ref. (0) |
| No | 2 940 | 0.49 (0.39 to 0.59)*** | 0.37 (0.27 to 0.47)*** | 0.33 (0.23 to 0.43)*** |
| **Hungry** | | | | |
| Never | 6 531 | Ref. (0) | Ref. (0) | Ref. (0) |
| At least once per year | 2 185 | 0.43 (0.32 to 0.54)*** | 0.35 (0.24 to 0.46)*** | 0.30 (0.19 to 0.41)*** |
| **Meat eating** | | | | |
| Daily | 1 119 | Ref. (0) | Ref. (0) | Ref. (0) |
| < Daily | 7 597 | 0.29 (0.15 to 0.43)*** | 0.15 (0.01 to 0.29)* | 0.12 (-0.02 to 0.26) |

CI, confidence interval; GDS, Geriatric Depression Scale: higher scores indicating more negative symptoms; N, number; Ref, reference.
Model 1: adjusting for sex, and age.
Model 2: additionally adjusting for education, occupation, marital status, household income, smoking, alcohol drinking, physical activity, body mass index, and stressful life events in adulthood.
Model 3: additionally adjusting for adverse childhood experiences (ACE score).
$^{*}P < 0.05$, $^{**}P < 0.01$, $^{***}P < 0.001$.

Table S2. Associations of each adverse childhood experience item with GDS score in adulthood.

| | *N* | Adjusted mean differences β (95% CI) in GDS score | | |
|---|---|---|---|---|
| | | Model 1 | Model 2 | Model 3 |
| **Separation from mother for more than one year continuously** | | | | |
| No | 6 627 | Ref. (0) | Ref. (0) | Ref. (0) |
| Yes | 2 089 | 0.39 (0.28 to 0.51)*** | 0.39 (0.28 to 0.50)*** | 0.40 (0.29 to 0.51)*** |
| **Frightening experience thought about years afterwards** | | | | |
| No | 7 854 | Ref. (0) | Ref. (0) | Ref. (0) |
| Yes | 862 | 0.97 (0.81 to 1.13)*** | 0.94 (0.79 to 1.10)*** | 0.91 (0.76 to 1.07)*** |
| **Sent away from home because of wrongdoing** | | | | |
| No | 8 525 | Ref. (0) | Ref. (0) | Ref. (0) |
| Yes | 191 | 0.88 (0.55 to 1.20)*** | 0.70 (0.38 to 1.01)*** | 0.66 (0.35 to 0.98)*** |
| **Parents quarrelling frequently** | | | | |
| No | 8 081 | Ref. (0) | Ref. (0) | Ref. (0) |
| Yes | 635 | 0.98 (0.80 to 1.16)*** | 0.90 (0.72 to 1.08)*** | 0.86 (0.68 to 1.04)*** |
| **Early parental death including one or two deaths** | | | | |
| No | 7 007 | Ref. (0) | Ref. (0) | Ref. (0) |
| Yes | 1 709 | 0.14 (0.02 to 0.26)* | 0.12 (-0.0004 to 0.24) | 0.09 (-0.03 to 0.21) |

CI: confidence interval; GDS, Geriatric Depression Scale: higher scores indicating more negative symptoms; N, number; Ref, reference.

Model 1: adjusting for sex, and age.

Model 2: additionally adjusting for education, occupation, marital status, household income, smoking, alcohol drinking, physical activity, body mass index, and stressful life events in adulthood.

Model 3: additionally adjusting for childhood socioeconomic disadvantages (CSD score).

*P <0.05, **P <0.01, ***P <0.001.

Table S3. Associations of childhood socioeconomic disadvantages and adverse childhood experiences with depressive symptoms in adulthood.

| | *N* (% cases) | Adjusted OR (95% CI) of depressive symptoms (GDS ≥ 8) | | |
|---|---|---|---|---|
| | | Model 1 | Model 2 | Model 3 |
| **Number of childhood socioeconomic disadvantage (CSD) items (score)** | | | | |
| 0 | 517 (2.51) | Ref. (1) | Ref. (1) | Ref. (1) |
| 1 | 1 215 (1.81) | 0.71 (0.36 to 1.43) | 0.66 (0.33 to 1.32) | 0.65 (0.32 to 1.31) |
| 2 | 751 (3.33) | 1.34 (0.68 to 2.65) | 1.18 (0.60 to 2.35) | 1.13 (0.57 to 2.25) |
| 3 | 848 (3.07) | 1.22 (0.62 to 2.41) | 1.06 (0.54 to 2.10) | 1.02 (0.51 to 2.02) |
| 4 | 2 085 (3.45) | 1.37 (0.75 to 2.50) | 1.14 (0.62 to 2.10) | 1.09 (0.59 to 2.01) |
| 5 | 1 469 (5.17) | 2.11 (1.15 to 3.85)* | 1.65 (0.89 to 3.07) | 1.49 (0.80 to 2.78) |
| 6 | 1 091 (6.05) | 2.48 (1.34 to 4.58)** | 1.96 (1.05 to 3.69)* | 1.85 (0.98 to 3.48) |
| 7 | 740 (7.03) | 2.90 (1.54 to 5.43)** | 2.18 (1.14 to 4.16)* | 1.96 (1.02 to 3.76)* |
| Per CSD score | 8 716 (4.04) | 1.22 (1.15 to 1.30)*** | 1.18 (1.11 to 1.26)*** | 1.17 (1.09 to 1.24)*** |
| P for trend | | <0.001 | <0.001 | <0.001 |
| **Number of adverse childhood experience (ACE) items (score)** | | | | |
| 0 | 4 822 (2.90) | Ref. (1) | Ref. (1) | Ref. (1) |
| 1 | 2 591 (4.94) | 1.71 (1.34 to 2.19)*** | 1.71 (1.33 to 2.19)*** | 1.66 (1.29 to 2.13)*** |
| 2 | 1 040 (5.29) | 1.81 (1.31 to 2.51)*** | 1.73 (1.24 to 2.41)** | 1.64 (1.18 to 2.28)** |
| 3 | 235 (9.36) | 3.39 (2.11 to 5.45)*** | 3.02 (1.86 to 4.89)*** | 2.77 (1.71 to 4.50)*** |
| 4 | 26 (23.08) | 9.35 (3.67 to 23.82)*** | 8.50 (3.26 to 22.08)*** | 7.90 (3.05 to 20.50)*** |
| 5 | 1 (100.00) | - | - | - |
| Per ACE score | 8 716 (4.04) | 1.49 (1.33 to 1.67)*** | 1.45 (1.29 to 1.63)*** | 1.42 (1.26 to 1.59)*** |
| P for trend | | <0.001 | <0.001 | <0.001 |

CI: confidence interval; GDS, Geriatric Depression Scale: higher scores indicating more negative symptoms; N, number; OR, odds ratio; Ref, reference.

Model 1: adjusting for sex, and age.

Model 2: additionally adjusting for education, occupation, marital status, household income, smoking, alcohol drinking, physical activity, body mass index, and stressful life events in adulthood.

Model 3: additionally adjusting for adverse childhood experiences (ACE score) or childhood socioeconomic disadvantages (CSD score).

$^{*}P < 0.05$, $^{**}P < 0.01$, $^{***}P < 0.001$.

Table S4. Associations of childhood socioeconomic conditions (good versus poor) with GDS score in adulthood by social deprivation index.

| | *N* | Adjusted mean differences β (95% CI) in GDS score | | |
|---|---|---|---|---|
| | | Model 1 | Model 2 | Model 3 |
| | | **Low social deprivation index (SDI) in adulthood (0-1)** | | |
| **Childhood socioeconomic conditions by number of childhood socioeconomic disadvantage items** | | | | |
| Good (0-3) | 2 722 | Ref. (0) | Ref. (0) | Ref. (0) |
| Poor (4-7) | 3 269 | 0.16 (0.05 to 0.27)** | 0.09 (-0.02 to 0.21) | 0.05 (-0.06 to 0.16) |
| | | **High social deprivation index (SDI) in adulthood (2-4)** | | |
| **Childhood socioeconomic conditions by number of childhood socioeconomic disadvantage items** | | | | |
| Good (0-3) | 267 | Ref. (0) | Ref. (0) | Ref. (0) |
| Poor (4-7) | 1 058 | 0.60 (0.24 to 0.97)** | 0.59 (0.22 to 0.96)** | 0.50 (0.13 to 0.86)** |
| P for SDI interaction | | 0.01 | 0.003 | 0.004 |

CI: confidence interval; GDS, Geriatric Depression Scale: higher scores indicating more negative symptoms; N, number; Ref, reference.

Model 1: adjusting for sex, and age.

Model 2: additionally adjusting for education, occupation, marital status, household income, smoking, alcohol drinking, physical activity, body mass index, and stressful life events in adulthood.

Model 3: additionally adjusting for adverse childhood experiences.

*P <0.05, **P <0.01, ***P <0.001.
